# Methyl 5-ferrocenyl-5a-hydr­oxy-1-methyl-10-oxo-2,3,3a,4,5a,10-hexa­hydro-1*H*-indeno[1,2:2′,3′]furo[3′,4′-*b*]pyrrole-3a-carboxyl­ate

**DOI:** 10.1107/S1600536809018583

**Published:** 2009-05-29

**Authors:** E. Theboral Sugi Kamala, S. Nirmala, L. Sudha, S. Kathiravan, R. Raghunathan

**Affiliations:** a Department of Physics, Easwari Engineering College, Ramapuram, Chennai 600 089, India; b Department of Physics, SRM University, Ramapuram Campus, Chennai 600 089, India; c Department of Organic Chemistry, University of Madras, Guindy Campus, Chennai 600 025, India

## Abstract

In the title compound, [Fe(C_5_H_5_)(C_21_H_20_NO_5_)], the pyrrolidine and cyclo­penta­none rings exhibit a twist conformation. The pyrrolidine ring is almost perpendicular to the cyclo­penta­none ring, making a dihedral angle of 81.91 (6)°. The mol­ecular conformation is stabilized by an intra­molecular O—H⋯N hydrogen bond and C—H⋯O inter­actions. The crystal structure is stabilized by inter­molecular C—H⋯O inter­actions.

## Related literature

For general background and uses of ferrocene-based ligands, see Gomez Arrayas *et al.* (2006 ▶); Blaser & Schmidt (2004 ▶); Johnson & Sames (2000 ▶); Baar *et al.* (2000 ▶); Staveren & Metzler-Nolte (2004 ▶). For puckering parameters, see: Cremer & Pople (1975 ▶). For asymmetry parameters, see: Nardelli (1983 ▶).
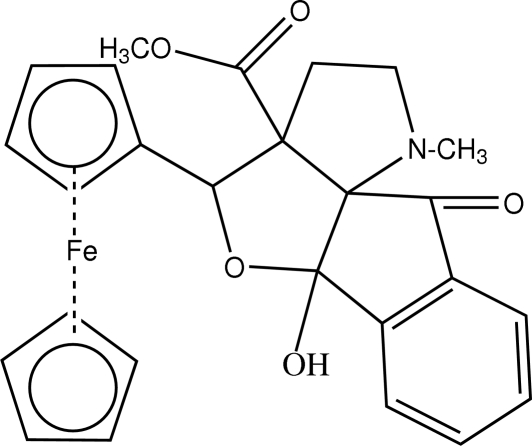


## Experimental

### 

#### Crystal data

[Fe(C_5_H_5_)(C_21_H_20_NO_5_)]
*M* *_r_* = 487.32
Monoclinic, 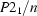

*a* = 7.7292 (2) Å
*b* = 24.7713 (7) Å
*c* = 11.8120 (4) Å
β = 93.4450 (10)°
*V* = 2257.47 (12) Å^3^
*Z* = 4
Mo *K*α radiation
μ = 0.71 mm^−1^
*T* = 293 K
0.30 × 0.25 × 0.20 mm

#### Data collection

Bruker Kappa APEXII diffractometer
Absorption correction: multi-scan (*SADABS*; Sheldrick, 1996 ▶) *T*_min_ = 0.816, *T*_max_ = 0.872
32884 measured reflections
7917 independent reflections
6016 reflections with *I* > 2σ(*I*)
*R*_int_ = 0.028

#### Refinement

*R*[*F* ^2^ > 2σ(*F* ^2^)] = 0.039
*wR*(*F* ^2^) = 0.123
*S* = 1.05
7917 reflections
299 parameters
H-atom parameters constrained
Δρ_max_ = 0.41 e Å^−3^
Δρ_min_ = −0.35 e Å^−3^

### 

Data collection: *APEX2* (Bruker, 2004 ▶); cell refinement: *APEX2* and *SAINT* (Bruker, 2004 ▶); data reduction: *APEX2* and *SAINT*; program(s) used to solve structure: *SIR92* (Altomare *et al.*, 1993 ▶); program(s) used to refine structure: *SHELXL97* (Sheldrick, 2008 ▶); molecular graphics: *ORTEP-3* (Farrugia, 1997 ▶); software used to prepare material for publication: *PLATON* (Spek, 2009 ▶).

## Supplementary Material

Crystal structure: contains datablocks I, global. DOI: 10.1107/S1600536809018583/bt2949sup1.cif

Structure factors: contains datablocks I. DOI: 10.1107/S1600536809018583/bt2949Isup2.hkl

Additional supplementary materials:  crystallographic information; 3D view; checkCIF report

## Figures and Tables

**Table 1:** Hydrogen-bond geometry (Å, °)

*D*—H⋯*A*	*D*—H	H⋯*A*	*D*⋯*A*	*D*—H⋯*A*
C7—H7⋯O4^i^	0.93	2.46	3.157 (2)	131
C15—H15⋯O3^ii^	0.98	2.52	3.303 (2)	137
C16—H16⋯O4^ii^	0.98	2.59	3.547 (2)	166
C13—H13⋯O5	0.98	2.40	2.820 (2)	105
C24—H24*B*⋯O3	0.96	2.42	3.014 (3)	120
O2—H2*C*⋯N1	0.82	2.15	2.6414 (19)	119
C2—H2*A*⋯O4	0.97	2.42	2.761 (2)	100

Symmetry codes: (i) 
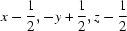
; (ii) 
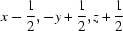
.

**Fig. 1.**
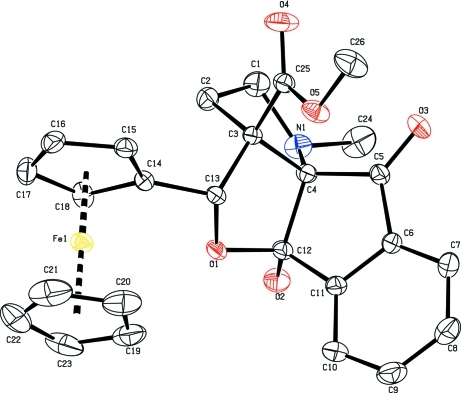
The molecular structure of (I) with 30% probability displacement ellipsoids.

**Fig. 2.**
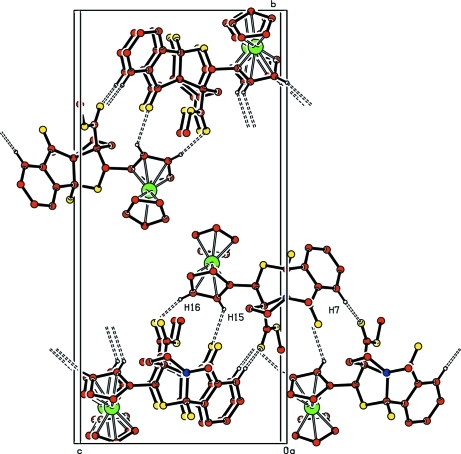
The packing of the molecules viewed along b axis.

## supplementary crystallographic information

### Comment

Ferrocene-based ligands incorporating chirality are very important (Gomez Arrayas *et al.*, 2006) and some of them have already been applied in industrial processes because of their stability, low price and unique structure (Blaser & Schmidt, 2004). Transition metal complexes derived from ferrocene have attracted great intrest due to their applications as precursors for the synthesis of organic as well as organometallic compounds (Johnson & Sames, 2000), in homogeneous catalysis (Baar *et al.*, 2000), or even in biological chemistry (Staveren & Metzler-Nolte, 2004).

Fig. 1 shows the *ORTEP* plot of compound (I). Bond lengths and angles are comparable with other reported values.

In the molecule the pyrrolidine ring N1/C1/C2/C3/C4 exhibits *twist* conformation with assymetry parameters (Nardelli, 1983) ΔC_s_(C1) = 12.47 (2)/ (C2) = 17.76 (21) and with the puckering parameters (Cremer & Pople, 1975) q2 = 0.3819 (2) Å and φ2 = 53.2 (2)°. The cyclopentanone ring also exhibits *twist* conformation with assymetry parameters ΔC_s_(C4) = 4.07 (2)/ (C12) = 4.11 (2) and with the puckering parameters q2 = 0.1106 (2) Å and φ2 = 343.6 (8)°. The sum of bond angles around N1 [341.70 (4)°] indicates *sp*^2^ hybridization. The pyrrolidine ring is almost perpendicular to the cyclopentanone ring making a dihedral angle of 81.91 (6)° and the ferrocene ring is perpendicular to the phenyl ring with a dihedral angle of 84.37 (8)°. The cyclopentanone and the phenyl rings are planar with each other making an angle of 5.06 (5)°.

In the crystal packing, atoms O3 and O4 are involved in intermolecular C—H···O interactions and atom O2 contributes to N—H···O intramolecular interactions.

### Experimental

A mixture of ferrocenyl Baylis–Hillman adduct, sarcosine and ninhydrin were refluxed in 1,2-dichloethane for 35 h and the solvent was removed under reduced pressure. The crude product was subjected to column chromatography to get the pure product. The product was recrystallized from dry benzene by slow evaporation.

### Refinement

H atoms were placed in idealized positions and allowed to ride on their parent atoms, with C—H = 0.93 or 0.96 Å and U_iso_(H)= 1.2–1.5U_eq_(C).

### Crystal data

**Table:**

[Fe(C_5_H_5_)(C_21_H_20_NO_5_)]	*F*(000) = 1016
*M**_r_* = 487.32	*D*_x_ = 1.434 Mg m^−^^3^
Monoclinic, *P*2_1_/*n*	Mo *K*α radiation, λ = 0.71073 Å
Hall symbol: -P2yn	Cell parameters from 32884 reflections
*a* = 7.7292 (2) Å	θ = 1.6–32.1°
*b* = 24.7713 (7) Å	µ = 0.71 mm^−^^1^
*c* = 11.8120 (4) Å	*T* = 293 K
β = 93.445 (1)°	Prism, orange
*V* = 2257.47 (12) Å^3^	0.30 × 0.25 × 0.20 mm
*Z* = 4	

### Data collection

**Table:**

Bruker Kappa APEXII diffractometer	7917 independent reflections
Radiation source: fine-focus sealed tube	6016 reflections with *I* > 2σ(*I*)
graphite	*R*_int_ = 0.028
ω and φ scans	θ_max_ = 32.1°, θ_min_ = 1.6°
Absorption correction: multi-scan (*SADABS*; Sheldrick, 1996)	*h* = −11→10
*T*_min_ = 0.816, *T*_max_ = 0.872	*k* = −37→34
32884 measured reflections	*l* = −17→17

### Refinement

**Table:**

Refinement on *F*^2^	Primary atom site location: structure-invariant direct methods
Least-squares matrix: full	Secondary atom site location: difference Fourier map
*R*[*F*^2^ > 2σ(*F*^2^)] = 0.039	Hydrogen site location: inferred from neighbouring sites
*wR*(*F*^2^) = 0.123	H-atom parameters constrained
*S* = 1.05	*w* = 1/[σ^2^(*F*_o_^2^) + (0.067*P*)^2^ + 0.4193*P*] where *P* = (*F*_o_^2^ + 2*F*_c_^2^)/3
7917 reflections	(Δ/σ)_max_ < 0.001
299 parameters	Δρ_max_ = 0.41 e Å^−^^3^
0 restraints	Δρ_min_ = −0.35 e Å^−^^3^

### Special details

**Table:**

Geometry. All e.s.d.'s (except the e.s.d. in the dihedral angle between two l.s. planes) are estimated using the full covariance matrix. The cell e.s.d.'s are taken into account individually in the estimation of e.s.d.'s in distances, angles and torsion angles; correlations between e.s.d.'s in cell parameters are only used when they are defined by crystal symmetry. An approximate (isotropic) treatment of cell e.s.d.'s is used for estimating e.s.d.'s involving l.s. planes.
Refinement. Refinement of *F*^2^ against ALL reflections. The weighted *R*-factor *wR* and goodness of fit *S* are based on *F*^2^, conventional *R*-factors *R* are based on *F*, with *F* set to zero for negative *F*^2^. The threshold expression of *F*^2^ > σ(*F*^2^) is used only for calculating *R*-factors(gt) *etc*. and is not relevant to the choice of reflections for refinement. *R*-factors based on *F*^2^ are statistically about twice as large as those based on *F*, and *R*- factors based on ALL data will be even larger.

### Fractional atomic coordinates and isotropic or equivalent isotropic displacement parameters (Å^2^)

**Table:**

	*x*	*y*	*z*	*U*_iso_*/*U*_eq_	
C1	0.4102 (2)	0.20020 (8)	0.59190 (15)	0.0407 (4)	
H1A	0.4053	0.2376	0.5678	0.049*	
H1B	0.5254	0.1924	0.6249	0.049*	
C2	0.2738 (2)	0.18825 (7)	0.67450 (13)	0.0346 (3)	
H2A	0.2634	0.2178	0.7276	0.042*	
H2B	0.3008	0.1554	0.7165	0.042*	
C3	0.10722 (17)	0.18175 (5)	0.59839 (11)	0.0248 (2)	
C4	0.17555 (18)	0.15859 (5)	0.48640 (11)	0.0270 (3)	
C5	0.0840 (2)	0.18021 (6)	0.37684 (11)	0.0305 (3)	
C6	−0.0361 (2)	0.13816 (6)	0.33231 (11)	0.0306 (3)	
C7	−0.1570 (2)	0.14203 (7)	0.24089 (13)	0.0412 (4)	
H7	−0.1660	0.1732	0.1970	0.049*	
C8	−0.2628 (3)	0.09837 (9)	0.21740 (16)	0.0499 (4)	
H8	−0.3440	0.0997	0.1561	0.060*	
C9	−0.2499 (3)	0.05245 (8)	0.28383 (17)	0.0520 (5)	
H9	−0.3239	0.0236	0.2668	0.062*	
C10	−0.1296 (3)	0.04837 (7)	0.37506 (16)	0.0437 (4)	
H10	−0.1222	0.0174	0.4195	0.052*	
C11	−0.0204 (2)	0.09189 (6)	0.39817 (12)	0.0304 (3)	
C12	0.12180 (19)	0.09758 (6)	0.48990 (11)	0.0288 (3)	
C13	−0.01516 (18)	0.13617 (5)	0.63622 (11)	0.0258 (2)	
H13	−0.1289	0.1401	0.5957	0.031*	
C14	−0.03749 (18)	0.13378 (6)	0.76052 (11)	0.0284 (3)	
C15	−0.1605 (2)	0.16454 (6)	0.81773 (13)	0.0346 (3)	
H15	−0.2452	0.1897	0.7822	0.042*	
C16	−0.1410 (3)	0.15208 (7)	0.93502 (13)	0.0428 (4)	
H16	−0.2098	0.1671	0.9946	0.051*	
C17	−0.0065 (2)	0.11379 (8)	0.95056 (13)	0.0451 (4)	
H17	0.0345	0.0976	1.0230	0.054*	
C18	0.0584 (2)	0.10234 (7)	0.84277 (13)	0.0367 (3)	
H18	0.1522	0.0772	0.8280	0.044*	
C19	−0.3003 (3)	0.02961 (9)	0.7276 (2)	0.0606 (6)	
H19	−0.2700	0.0262	0.6485	0.073*	
C20	−0.4307 (3)	0.06263 (10)	0.7669 (2)	0.0666 (6)	
H20	−0.5094	0.0859	0.7208	0.080*	
C21	−0.4295 (3)	0.05528 (12)	0.8862 (3)	0.0791 (9)	
H21	−0.5074	0.0728	0.9373	0.095*	
C22	−0.2990 (4)	0.01844 (11)	0.9176 (2)	0.0767 (8)	
H22	−0.2686	0.0056	0.9948	0.092*	
C23	−0.2207 (4)	0.00267 (9)	0.8196 (2)	0.0687 (7)	
H23	−0.1242	−0.0228	0.8164	0.082*	
C24	0.4551 (3)	0.17375 (11)	0.39497 (19)	0.0616 (6)	
H24A	0.5773	0.1763	0.4142	0.074*	
H24B	0.4149	0.2069	0.3607	0.074*	
H24C	0.4335	0.1446	0.3426	0.074*	
C25	0.01735 (19)	0.23602 (6)	0.58256 (12)	0.0295 (3)	
C26	−0.2250 (3)	0.28321 (8)	0.5066 (2)	0.0582 (5)	
H26A	−0.3369	0.2767	0.4694	0.070*	
H26B	−0.1564	0.3044	0.4581	0.070*	
H26C	−0.2385	0.3023	0.5762	0.070*	
N1	0.36358 (17)	0.16382 (6)	0.49745 (12)	0.0385 (3)	
O1	0.06469 (15)	0.08784 (4)	0.60056 (9)	0.0320 (2)	
O2	0.25662 (16)	0.06304 (5)	0.47192 (10)	0.0409 (3)	
H2C	0.3484	0.0773	0.4936	0.049*	
O3	0.10650 (19)	0.22437 (5)	0.33645 (11)	0.0487 (3)	
O4	0.07972 (19)	0.27824 (5)	0.61256 (14)	0.0553 (4)	
O5	−0.13909 (15)	0.23198 (5)	0.53094 (11)	0.0406 (3)	
Fe1	−0.19791 (3)	0.084076 (8)	0.842289 (18)	0.03124 (7)	

### Atomic displacement parameters (Å^2^)

**Table:**

	*U*^11^	*U*^22^	*U*^33^	*U*^12^	*U*^13^	*U*^23^
C1	0.0290 (7)	0.0467 (9)	0.0460 (8)	−0.0073 (7)	−0.0002 (6)	−0.0006 (7)
C2	0.0332 (7)	0.0373 (8)	0.0327 (7)	−0.0035 (6)	−0.0035 (5)	0.0020 (6)
C3	0.0276 (6)	0.0219 (6)	0.0249 (5)	−0.0009 (5)	0.0025 (4)	−0.0005 (4)
C4	0.0298 (6)	0.0230 (6)	0.0287 (6)	−0.0015 (5)	0.0054 (5)	−0.0003 (5)
C5	0.0382 (7)	0.0266 (6)	0.0275 (6)	−0.0022 (6)	0.0072 (5)	0.0023 (5)
C6	0.0387 (7)	0.0281 (7)	0.0253 (6)	−0.0013 (6)	0.0054 (5)	−0.0010 (5)
C7	0.0518 (10)	0.0428 (9)	0.0287 (7)	0.0018 (7)	−0.0007 (6)	0.0008 (6)
C8	0.0514 (11)	0.0580 (11)	0.0392 (8)	−0.0047 (9)	−0.0072 (7)	−0.0075 (8)
C9	0.0543 (11)	0.0458 (10)	0.0553 (11)	−0.0171 (9)	−0.0026 (9)	−0.0117 (8)
C10	0.0570 (10)	0.0286 (8)	0.0456 (9)	−0.0097 (7)	0.0026 (8)	−0.0012 (6)
C11	0.0386 (7)	0.0245 (6)	0.0286 (6)	−0.0016 (5)	0.0053 (5)	−0.0024 (5)
C12	0.0372 (7)	0.0209 (6)	0.0285 (6)	0.0023 (5)	0.0046 (5)	−0.0001 (5)
C13	0.0297 (6)	0.0206 (6)	0.0273 (6)	−0.0018 (5)	0.0029 (5)	0.0024 (4)
C14	0.0318 (7)	0.0268 (6)	0.0269 (6)	−0.0043 (5)	0.0036 (5)	0.0020 (5)
C15	0.0434 (8)	0.0256 (7)	0.0355 (7)	−0.0013 (6)	0.0077 (6)	−0.0029 (5)
C16	0.0577 (10)	0.0400 (9)	0.0316 (7)	−0.0117 (8)	0.0108 (7)	−0.0087 (6)
C17	0.0525 (10)	0.0547 (11)	0.0278 (7)	−0.0113 (8)	−0.0008 (6)	0.0069 (7)
C18	0.0320 (7)	0.0431 (9)	0.0349 (7)	0.0003 (6)	0.0019 (6)	0.0098 (6)
C19	0.0717 (14)	0.0498 (12)	0.0620 (12)	−0.0267 (10)	0.0180 (10)	−0.0215 (10)
C20	0.0466 (11)	0.0551 (13)	0.0970 (19)	−0.0138 (10)	−0.0048 (11)	−0.0154 (12)
C21	0.0657 (15)	0.0739 (17)	0.103 (2)	−0.0361 (13)	0.0521 (15)	−0.0357 (15)
C22	0.103 (2)	0.0593 (14)	0.0700 (15)	−0.0363 (15)	0.0245 (14)	0.0153 (11)
C23	0.0789 (16)	0.0283 (9)	0.1009 (19)	−0.0077 (10)	0.0216 (14)	−0.0013 (10)
C24	0.0436 (10)	0.0817 (16)	0.0623 (12)	−0.0127 (10)	0.0264 (9)	−0.0172 (11)
C25	0.0339 (7)	0.0238 (6)	0.0311 (6)	−0.0002 (5)	0.0056 (5)	0.0002 (5)
C26	0.0475 (11)	0.0378 (10)	0.0881 (16)	0.0124 (8)	−0.0049 (10)	0.0137 (10)
N1	0.0281 (6)	0.0417 (8)	0.0465 (7)	−0.0027 (5)	0.0093 (5)	−0.0054 (6)
O1	0.0481 (6)	0.0197 (4)	0.0289 (5)	0.0020 (4)	0.0072 (4)	0.0034 (3)
O2	0.0459 (7)	0.0315 (6)	0.0457 (6)	0.0122 (5)	0.0054 (5)	−0.0037 (5)
O3	0.0688 (9)	0.0340 (6)	0.0431 (6)	−0.0140 (6)	0.0023 (6)	0.0134 (5)
O4	0.0585 (8)	0.0255 (6)	0.0798 (10)	−0.0030 (6)	−0.0130 (7)	−0.0090 (6)
O5	0.0344 (6)	0.0277 (5)	0.0590 (7)	0.0032 (4)	−0.0034 (5)	0.0062 (5)
Fe1	0.03617 (13)	0.02629 (11)	0.03231 (11)	−0.00393 (8)	0.01072 (8)	−0.00053 (7)

### Geometric parameters (Å, °)

**Table:**

C1—N1	1.462 (2)	C15—H15	0.9800
C1—C2	1.508 (2)	C16—C17	1.411 (3)
C1—H1A	0.9700	C16—Fe1	2.0428 (16)
C1—H1B	0.9700	C16—H16	0.9800
C2—C3	1.534 (2)	C17—C18	1.425 (2)
C2—H2A	0.9700	C17—Fe1	2.0342 (18)
C2—H2B	0.9700	C17—H17	0.9800
C3—C25	1.5195 (19)	C18—Fe1	2.0315 (16)
C3—C13	1.5557 (18)	C18—H18	0.9800
C3—C4	1.5626 (18)	C19—C23	1.388 (4)
C4—N1	1.4571 (19)	C19—C20	1.399 (3)
C4—C5	1.534 (2)	C19—Fe1	2.0380 (19)
C4—C12	1.5686 (19)	C19—H19	0.9800
C5—O3	1.2102 (18)	C20—C21	1.419 (4)
C5—C6	1.472 (2)	C20—Fe1	2.029 (2)
C6—C11	1.386 (2)	C20—H20	0.9800
C6—C7	1.388 (2)	C21—C22	1.394 (4)
C7—C8	1.374 (3)	C21—Fe1	2.023 (2)
C7—H7	0.9300	C21—H21	0.9800
C8—C9	1.382 (3)	C22—C23	1.394 (4)
C8—H8	0.9300	C22—Fe1	2.032 (2)
C9—C10	1.384 (3)	C22—H22	0.9800
C9—H9	0.9300	C23—Fe1	2.040 (2)
C10—C11	1.386 (2)	C23—H23	0.9800
C10—H10	0.9300	C24—N1	1.459 (2)
C11—C12	1.503 (2)	C24—H24A	0.9600
C12—O2	1.3747 (18)	C24—H24B	0.9600
C12—O1	1.4251 (17)	C24—H24C	0.9600
C13—O1	1.4223 (16)	C25—O4	1.1963 (19)
C13—C14	1.4897 (18)	C25—O5	1.3248 (19)
C13—H13	0.9800	C26—O5	1.453 (2)
C14—C18	1.419 (2)	C26—H26A	0.9600
C14—C15	1.421 (2)	C26—H26B	0.9600
C14—Fe1	2.0323 (13)	C26—H26C	0.9600
C15—C16	1.418 (2)	O2—H2C	0.8200
C15—Fe1	2.0372 (15)		
			
N1—C1—C2	102.88 (13)	Fe1—C19—H19	125.8
N1—C1—H1A	111.2	C19—C20—C21	107.0 (2)
C2—C1—H1A	111.2	C19—C20—Fe1	70.21 (13)
N1—C1—H1B	111.2	C21—C20—Fe1	69.27 (13)
C2—C1—H1B	111.2	C19—C20—H20	126.5
H1A—C1—H1B	109.1	C21—C20—H20	126.5
C1—C2—C3	103.66 (12)	Fe1—C20—H20	126.5
C1—C2—H2A	111.0	C22—C21—C20	108.1 (2)
C3—C2—H2A	111.0	C22—C21—Fe1	70.23 (13)
C1—C2—H2B	111.0	C20—C21—Fe1	69.73 (12)
C3—C2—H2B	111.0	C22—C21—H21	126.0
H2A—C2—H2B	109.0	C20—C21—H21	126.0
C25—C3—C2	109.92 (12)	Fe1—C21—H21	126.0
C25—C3—C13	113.33 (11)	C23—C22—C21	107.8 (2)
C2—C3—C13	114.28 (11)	C23—C22—Fe1	70.31 (12)
C25—C3—C4	113.32 (11)	C21—C22—Fe1	69.56 (13)
C2—C3—C4	102.72 (11)	C23—C22—H22	126.1
C13—C3—C4	102.65 (10)	C21—C22—H22	126.1
N1—C4—C5	116.85 (12)	Fe1—C22—H22	126.1
N1—C4—C3	106.11 (11)	C19—C23—C22	108.8 (2)
C5—C4—C3	115.08 (11)	C19—C23—Fe1	70.02 (12)
N1—C4—C12	110.34 (12)	C22—C23—Fe1	69.67 (13)
C5—C4—C12	104.43 (11)	C19—C23—H23	125.6
C3—C4—C12	103.17 (10)	C22—C23—H23	125.6
O3—C5—C6	126.98 (14)	Fe1—C23—H23	125.6
O3—C5—C4	125.34 (14)	N1—C24—H24A	109.5
C6—C5—C4	107.67 (11)	N1—C24—H24B	109.5
C11—C6—C7	121.73 (15)	H24A—C24—H24B	109.5
C11—C6—C5	110.64 (13)	N1—C24—H24C	109.5
C7—C6—C5	127.58 (14)	H24A—C24—H24C	109.5
C8—C7—C6	117.86 (16)	H24B—C24—H24C	109.5
C8—C7—H7	121.1	O4—C25—O5	122.86 (15)
C6—C7—H7	121.1	O4—C25—C3	124.32 (14)
C7—C8—C9	120.77 (17)	O5—C25—C3	112.81 (12)
C7—C8—H8	119.6	O5—C26—H26A	109.5
C9—C8—H8	119.6	O5—C26—H26B	109.5
C8—C9—C10	121.61 (17)	H26A—C26—H26B	109.5
C8—C9—H9	119.2	O5—C26—H26C	109.5
C10—C9—H9	119.2	H26A—C26—H26C	109.5
C9—C10—C11	117.98 (16)	H26B—C26—H26C	109.5
C9—C10—H10	121.0	C4—N1—C24	118.13 (15)
C11—C10—H10	121.0	C4—N1—C1	108.77 (12)
C10—C11—C6	120.03 (15)	C24—N1—C1	114.80 (15)
C10—C11—C12	128.69 (14)	C13—O1—C12	107.04 (10)
C6—C11—C12	111.27 (13)	C12—O2—H2C	109.5
O2—C12—O1	108.41 (11)	C25—O5—C26	114.76 (14)
O2—C12—C11	111.00 (12)	C21—Fe1—C20	41.00 (12)
O1—C12—C11	113.09 (12)	C21—Fe1—C18	162.82 (11)
O2—C12—C4	113.03 (12)	C20—Fe1—C18	153.91 (9)
O1—C12—C4	106.52 (10)	C21—Fe1—C22	40.21 (13)
C11—C12—C4	104.76 (11)	C20—Fe1—C22	68.19 (12)
O1—C13—C14	109.66 (11)	C18—Fe1—C22	125.27 (11)
O1—C13—C3	104.10 (10)	C21—Fe1—C14	155.48 (12)
C14—C13—C3	114.86 (11)	C20—Fe1—C14	120.02 (9)
O1—C13—H13	109.3	C18—Fe1—C14	40.87 (6)
C14—C13—H13	109.3	C22—Fe1—C14	162.65 (11)
C3—C13—H13	109.3	C21—Fe1—C17	126.00 (10)
C18—C14—C15	107.79 (13)	C20—Fe1—C17	164.24 (10)
C18—C14—C13	127.59 (13)	C18—Fe1—C17	41.03 (7)
C15—C14—C13	124.61 (13)	C22—Fe1—C17	107.36 (10)
C18—C14—Fe1	69.53 (8)	C14—Fe1—C17	68.79 (6)
C15—C14—Fe1	69.76 (8)	C21—Fe1—C15	121.12 (10)
C13—C14—Fe1	127.05 (10)	C20—Fe1—C15	108.87 (9)
C16—C15—C14	108.25 (14)	C18—Fe1—C15	68.64 (7)
C16—C15—Fe1	69.87 (9)	C22—Fe1—C15	155.06 (10)
C14—C15—Fe1	69.39 (8)	C14—Fe1—C15	40.86 (6)
C16—C15—H15	125.9	C17—Fe1—C15	68.37 (7)
C14—C15—H15	125.9	C21—Fe1—C19	67.84 (10)
Fe1—C15—H15	125.9	C20—Fe1—C19	40.24 (10)
C17—C16—C15	107.91 (14)	C18—Fe1—C19	119.26 (8)
C17—C16—Fe1	69.42 (10)	C22—Fe1—C19	67.50 (11)
C15—C16—Fe1	69.44 (9)	C14—Fe1—C19	107.98 (8)
C17—C16—H16	126.0	C17—Fe1—C19	153.65 (10)
C15—C16—H16	126.0	C15—Fe1—C19	127.29 (9)
Fe1—C16—H16	126.0	C21—Fe1—C23	67.31 (11)
C16—C17—C18	108.28 (14)	C20—Fe1—C23	67.44 (11)
C16—C17—Fe1	70.08 (10)	C18—Fe1—C23	107.28 (9)
C18—C17—Fe1	69.39 (9)	C22—Fe1—C23	40.03 (11)
C16—C17—H17	125.9	C14—Fe1—C23	125.93 (8)
C18—C17—H17	125.9	C17—Fe1—C23	119.59 (10)
Fe1—C17—H17	125.9	C15—Fe1—C23	163.76 (9)
C14—C18—C17	107.77 (15)	C19—Fe1—C23	39.78 (11)
C14—C18—Fe1	69.60 (9)	C21—Fe1—C16	108.59 (9)
C17—C18—Fe1	69.59 (10)	C20—Fe1—C16	127.42 (9)
C14—C18—H18	126.1	C18—Fe1—C16	68.68 (8)
C17—C18—H18	126.1	C22—Fe1—C16	120.07 (9)
Fe1—C18—H18	126.1	C14—Fe1—C16	68.74 (6)
C23—C19—C20	108.3 (2)	C17—Fe1—C16	40.50 (8)
C23—C19—Fe1	70.20 (13)	C15—Fe1—C16	40.69 (6)
C20—C19—Fe1	69.55 (12)	C19—Fe1—C16	164.77 (10)
C23—C19—H19	125.8	C23—Fe1—C16	154.08 (10)
C20—C19—H19	125.8		
			
N1—C1—C2—C3	−40.15 (16)	C21—C20—Fe1—C19	118.0 (2)
C1—C2—C3—C25	−89.90 (14)	C19—C20—Fe1—C23	−37.12 (15)
C1—C2—C3—C13	141.37 (13)	C21—C20—Fe1—C23	80.86 (18)
C1—C2—C3—C4	31.00 (15)	C19—C20—Fe1—C16	167.58 (13)
C25—C3—C4—N1	107.73 (13)	C21—C20—Fe1—C16	−74.44 (19)
C2—C3—C4—N1	−10.81 (14)	C14—C18—Fe1—C21	−165.8 (3)
C13—C3—C4—N1	−129.67 (11)	C17—C18—Fe1—C21	−46.7 (3)
C25—C3—C4—C5	−23.13 (16)	C14—C18—Fe1—C20	51.6 (2)
C2—C3—C4—C5	−141.67 (12)	C17—C18—Fe1—C20	170.7 (2)
C13—C3—C4—C5	99.47 (13)	C14—C18—Fe1—C22	165.77 (12)
C25—C3—C4—C12	−136.22 (12)	C17—C18—Fe1—C22	−75.16 (15)
C2—C3—C4—C12	105.25 (12)	C17—C18—Fe1—C14	119.07 (15)
C13—C3—C4—C12	−13.62 (13)	C14—C18—Fe1—C17	−119.07 (15)
N1—C4—C5—O3	−49.5 (2)	C14—C18—Fe1—C15	−37.90 (9)
C3—C4—C5—O3	75.95 (19)	C17—C18—Fe1—C15	81.18 (11)
C12—C4—C5—O3	−171.71 (15)	C14—C18—Fe1—C19	83.87 (13)
N1—C4—C5—C6	131.42 (13)	C17—C18—Fe1—C19	−157.05 (13)
C3—C4—C5—C6	−103.11 (13)	C14—C18—Fe1—C23	125.43 (12)
C12—C4—C5—C6	9.24 (15)	C17—C18—Fe1—C23	−115.49 (13)
O3—C5—C6—C11	177.09 (16)	C14—C18—Fe1—C16	−81.73 (10)
C4—C5—C6—C11	−3.88 (16)	C17—C18—Fe1—C16	37.34 (11)
O3—C5—C6—C7	−5.5 (3)	C23—C22—Fe1—C21	118.6 (3)
C4—C5—C6—C7	173.49 (15)	C23—C22—Fe1—C20	80.45 (19)
C11—C6—C7—C8	0.4 (2)	C21—C22—Fe1—C20	−38.20 (16)
C5—C6—C7—C8	−176.70 (16)	C23—C22—Fe1—C18	−74.0 (2)
C6—C7—C8—C9	0.7 (3)	C21—C22—Fe1—C18	167.40 (14)
C7—C8—C9—C10	−0.8 (3)	C23—C22—Fe1—C14	−41.3 (4)
C8—C9—C10—C11	−0.3 (3)	C21—C22—Fe1—C14	−160.0 (2)
C9—C10—C11—C6	1.4 (3)	C23—C22—Fe1—C17	−115.62 (18)
C9—C10—C11—C12	−178.98 (17)	C21—C22—Fe1—C17	125.74 (16)
C7—C6—C11—C10	−1.5 (2)	C23—C22—Fe1—C15	168.48 (19)
C5—C6—C11—C10	176.04 (15)	C21—C22—Fe1—C15	49.8 (3)
C7—C6—C11—C12	178.83 (14)	C23—C22—Fe1—C19	36.85 (17)
C5—C6—C11—C12	−3.63 (17)	C21—C22—Fe1—C19	−81.80 (17)
C10—C11—C12—O2	67.4 (2)	C21—C22—Fe1—C23	−118.6 (3)
C6—C11—C12—O2	−112.98 (14)	C23—C22—Fe1—C16	−157.91 (16)
C10—C11—C12—O1	−54.7 (2)	C21—C22—Fe1—C16	83.44 (18)
C6—C11—C12—O1	124.92 (13)	C18—C14—Fe1—C21	169.91 (19)
C10—C11—C12—C4	−170.31 (16)	C15—C14—Fe1—C21	50.9 (2)
C6—C11—C12—C4	9.33 (16)	C13—C14—Fe1—C21	−67.8 (2)
N1—C4—C12—O2	−16.34 (16)	C18—C14—Fe1—C20	−156.55 (12)
C5—C4—C12—O2	110.01 (13)	C15—C14—Fe1—C20	84.43 (12)
C3—C4—C12—O2	−129.34 (12)	C13—C14—Fe1—C20	−34.27 (16)
N1—C4—C12—O1	102.61 (13)	C15—C14—Fe1—C18	−119.02 (13)
C5—C4—C12—O1	−131.04 (12)	C13—C14—Fe1—C18	122.28 (17)
C3—C4—C12—O1	−10.39 (14)	C18—C14—Fe1—C22	−42.3 (3)
N1—C4—C12—C11	−137.32 (12)	C15—C14—Fe1—C22	−161.3 (3)
C5—C4—C12—C11	−10.96 (14)	C13—C14—Fe1—C22	80.0 (3)
C3—C4—C12—C11	109.68 (12)	C18—C14—Fe1—C17	37.98 (11)
C25—C3—C13—O1	156.23 (11)	C15—C14—Fe1—C17	−81.04 (10)
C2—C3—C13—O1	−76.78 (13)	C13—C14—Fe1—C17	160.26 (15)
C4—C3—C13—O1	33.64 (12)	C18—C14—Fe1—C15	119.02 (13)
C25—C3—C13—C14	−83.85 (14)	C13—C14—Fe1—C15	−118.70 (16)
C2—C3—C13—C14	43.14 (16)	C18—C14—Fe1—C19	−114.22 (12)
C4—C3—C13—C14	153.56 (12)	C15—C14—Fe1—C19	126.75 (11)
O1—C13—C14—C18	23.8 (2)	C13—C14—Fe1—C19	8.06 (15)
C3—C13—C14—C18	−92.97 (18)	C18—C14—Fe1—C23	−73.92 (14)
O1—C13—C14—C15	−157.47 (14)	C15—C14—Fe1—C23	167.05 (13)
C3—C13—C14—C15	85.74 (17)	C13—C14—Fe1—C23	48.36 (18)
O1—C13—C14—Fe1	−67.80 (15)	C18—C14—Fe1—C16	81.57 (11)
C3—C13—C14—Fe1	175.41 (10)	C15—C14—Fe1—C16	−37.45 (10)
C18—C14—C15—C16	−0.10 (18)	C13—C14—Fe1—C16	−156.15 (15)
C13—C14—C15—C16	−179.03 (13)	C16—C17—Fe1—C21	−75.88 (16)
Fe1—C14—C15—C16	59.25 (11)	C18—C17—Fe1—C21	164.60 (14)
C18—C14—C15—Fe1	−59.36 (11)	C16—C17—Fe1—C20	−45.2 (4)
C13—C14—C15—Fe1	121.72 (14)	C18—C17—Fe1—C20	−164.8 (3)
C14—C15—C16—C17	0.01 (19)	C16—C17—Fe1—C18	119.52 (14)
Fe1—C15—C16—C17	58.96 (12)	C16—C17—Fe1—C22	−116.25 (13)
C14—C15—C16—Fe1	−58.95 (11)	C18—C17—Fe1—C22	124.22 (13)
C15—C16—C17—C18	0.1 (2)	C16—C17—Fe1—C14	81.68 (10)
Fe1—C16—C17—C18	59.06 (12)	C18—C17—Fe1—C14	−37.84 (10)
C15—C16—C17—Fe1	−58.97 (12)	C16—C17—Fe1—C15	37.64 (10)
C15—C14—C18—C17	0.16 (18)	C18—C17—Fe1—C15	−81.88 (11)
C13—C14—C18—C17	179.04 (14)	C16—C17—Fe1—C19	169.54 (17)
Fe1—C14—C18—C17	−59.34 (12)	C18—C17—Fe1—C19	50.0 (2)
C15—C14—C18—Fe1	59.49 (11)	C16—C17—Fe1—C23	−158.08 (11)
C13—C14—C18—Fe1	−121.62 (15)	C18—C17—Fe1—C23	82.39 (13)
C16—C17—C18—C14	−0.2 (2)	C18—C17—Fe1—C16	−119.52 (14)
Fe1—C17—C18—C14	59.34 (11)	C16—C15—Fe1—C21	82.47 (15)
C16—C17—C18—Fe1	−59.49 (13)	C14—C15—Fe1—C21	−157.90 (12)
C23—C19—C20—C21	0.0 (2)	C16—C15—Fe1—C20	125.98 (13)
Fe1—C19—C20—C21	−59.75 (15)	C14—C15—Fe1—C20	−114.40 (11)
C23—C19—C20—Fe1	59.70 (16)	C16—C15—Fe1—C18	−81.72 (11)
C19—C20—C21—C22	0.3 (3)	C14—C15—Fe1—C18	37.91 (9)
Fe1—C20—C21—C22	−60.03 (17)	C16—C15—Fe1—C22	47.3 (3)
C19—C20—C21—Fe1	60.35 (15)	C14—C15—Fe1—C22	166.9 (2)
C20—C21—C22—C23	−0.5 (3)	C16—C15—Fe1—C14	−119.63 (14)
Fe1—C21—C22—C23	−60.19 (16)	C16—C15—Fe1—C17	−37.47 (11)
C20—C21—C22—Fe1	59.72 (16)	C14—C15—Fe1—C17	82.15 (10)
C20—C19—C23—C22	−0.2 (3)	C16—C15—Fe1—C19	167.07 (12)
Fe1—C19—C23—C22	59.05 (17)	C14—C15—Fe1—C19	−73.31 (13)
C20—C19—C23—Fe1	−59.30 (15)	C16—C15—Fe1—C23	−160.1 (3)
C21—C22—C23—C19	0.4 (3)	C14—C15—Fe1—C23	−40.4 (4)
Fe1—C22—C23—C19	−59.27 (16)	C14—C15—Fe1—C16	119.63 (14)
C21—C22—C23—Fe1	59.71 (16)	C23—C19—Fe1—C21	−80.70 (18)
C2—C3—C25—O4	9.2 (2)	C20—C19—Fe1—C21	38.73 (18)
C13—C3—C25—O4	138.41 (17)	C23—C19—Fe1—C20	−119.4 (2)
C4—C3—C25—O4	−105.12 (18)	C23—C19—Fe1—C18	81.91 (15)
C2—C3—C25—O5	−171.14 (12)	C20—C19—Fe1—C18	−158.67 (15)
C13—C3—C25—O5	−41.90 (16)	C23—C19—Fe1—C22	−37.07 (16)
C4—C3—C25—O5	74.57 (15)	C20—C19—Fe1—C22	82.36 (18)
C5—C4—N1—C24	−17.7 (2)	C23—C19—Fe1—C14	125.06 (14)
C3—C4—N1—C24	−147.54 (16)	C20—C19—Fe1—C14	−115.51 (16)
C12—C4—N1—C24	101.35 (17)	C23—C19—Fe1—C17	46.7 (2)
C5—C4—N1—C1	115.47 (15)	C20—C19—Fe1—C17	166.12 (17)
C3—C4—N1—C1	−14.38 (16)	C23—C19—Fe1—C15	166.27 (13)
C12—C4—N1—C1	−125.48 (13)	C20—C19—Fe1—C15	−74.30 (18)
C2—C1—N1—C4	34.20 (17)	C20—C19—Fe1—C23	119.4 (2)
C2—C1—N1—C24	169.08 (16)	C23—C19—Fe1—C16	−160.0 (3)
C14—C13—O1—C12	−165.76 (11)	C20—C19—Fe1—C16	−40.6 (4)
C3—C13—O1—C12	−42.39 (13)	C19—C23—Fe1—C21	82.13 (17)
O2—C12—O1—C13	155.22 (12)	C22—C23—Fe1—C21	−37.89 (19)
C11—C12—O1—C13	−81.24 (14)	C19—C23—Fe1—C20	37.54 (15)
C4—C12—O1—C13	33.29 (14)	C22—C23—Fe1—C20	−82.5 (2)
O4—C25—O5—C26	3.9 (2)	C19—C23—Fe1—C18	−115.24 (14)
C3—C25—O5—C26	−175.77 (15)	C22—C23—Fe1—C18	124.75 (18)
C22—C21—Fe1—C20	118.9 (2)	C19—C23—Fe1—C22	120.0 (2)
C22—C21—Fe1—C18	−37.1 (4)	C19—C23—Fe1—C14	−74.06 (16)
C20—C21—Fe1—C18	−156.0 (3)	C22—C23—Fe1—C14	165.93 (16)
C20—C21—Fe1—C22	−118.9 (2)	C19—C23—Fe1—C17	−158.19 (13)
C22—C21—Fe1—C14	165.75 (18)	C22—C23—Fe1—C17	81.80 (19)
C20—C21—Fe1—C14	46.8 (3)	C19—C23—Fe1—C15	−42.5 (4)
C22—C21—Fe1—C17	−73.26 (18)	C22—C23—Fe1—C15	−162.5 (3)
C20—C21—Fe1—C17	167.82 (14)	C22—C23—Fe1—C19	−120.0 (2)
C22—C21—Fe1—C15	−157.89 (14)	C19—C23—Fe1—C16	168.13 (17)
C20—C21—Fe1—C15	83.18 (16)	C22—C23—Fe1—C16	48.1 (3)
C22—C21—Fe1—C19	80.90 (17)	C17—C16—Fe1—C21	124.13 (14)
C20—C21—Fe1—C19	−38.03 (15)	C15—C16—Fe1—C21	−116.43 (15)
C22—C21—Fe1—C23	37.71 (17)	C17—C16—Fe1—C20	165.95 (13)
C20—C21—Fe1—C23	−81.21 (17)	C15—C16—Fe1—C20	−74.62 (15)
C22—C21—Fe1—C16	−114.90 (16)	C17—C16—Fe1—C18	−37.82 (10)
C20—C21—Fe1—C16	126.18 (15)	C15—C16—Fe1—C18	81.62 (11)
C19—C20—Fe1—C21	−118.0 (2)	C17—C16—Fe1—C22	81.54 (15)
C19—C20—Fe1—C18	46.2 (3)	C15—C16—Fe1—C22	−159.02 (14)
C21—C20—Fe1—C18	164.15 (19)	C17—C16—Fe1—C14	−81.83 (10)
C19—C20—Fe1—C22	−80.49 (17)	C15—C16—Fe1—C14	37.61 (10)
C21—C20—Fe1—C22	37.49 (16)	C15—C16—Fe1—C17	119.44 (14)
C19—C20—Fe1—C14	82.48 (16)	C17—C16—Fe1—C15	−119.44 (14)
C21—C20—Fe1—C14	−159.54 (15)	C17—C16—Fe1—C19	−162.1 (3)
C19—C20—Fe1—C17	−156.9 (3)	C15—C16—Fe1—C19	−42.7 (3)
C21—C20—Fe1—C17	−38.9 (4)	C17—C16—Fe1—C23	48.0 (2)
C19—C20—Fe1—C15	125.96 (14)	C15—C16—Fe1—C23	167.39 (19)
C21—C20—Fe1—C15	−116.06 (16)		

### Hydrogen-bond geometry (Å, °)

**Table:**

*D*—H···*A*	*D*—H	H···*A*	*D*···*A*	*D*—H···*A*
C7—H7···O4^i^	0.93	2.46	3.157 (2)	131
C15—H15···O3^ii^	0.98	2.52	3.303 (2)	137
C16—H16···O4^ii^	0.98	2.59	3.547 (2)	166
C13—H13···O5	0.98	2.40	2.820 (2)	105
C24—H24B···O3	0.96	2.42	3.014 (3)	120
O2—H2C···N1	0.82	2.15	2.6414 (19)	119
C2—H2A···O4	0.97	2.42	2.761 (2)	100

Symmetry codes: (i) *x*−1/2, −*y*+1/2, *z*−1/2; (ii) *x*−1/2, −*y*+1/2, *z*+1/2.

## References

[bb1] Altomare, A., Cascarano, G., Giacovazzo, C. & Guagliardi, A. (1993). *J. Appl. Cryst.* **26**, 343–350.

[bb2] Baar, C. R., Carbray, L. P., Jennings, M. C. & Puddephatt, R. J. (2000). *J. Am. Chem. Soc* **122**, 176–177.

[bb3] Blaser, H. U. & Schmidt, E. (2004). In *Asymmetric Catalysis on Industrial Scale* Weinheim: Wiley–VCH.

[bb4] Bruker (2004). *APEX2* and *SAINT* Bruker AXS Inc., Madison, Wisconsin, USA.

[bb5] Cremer, D. & Pople, J. A. (1975). *J. Am. Chem. Soc.* **97**, 1354–1358.

[bb6] Farrugia, L. J. (1997). *J. Appl. Cryst.* **30**, 565.

[bb7] Gomez Arrayas, R., Adrio, J. & Carretero, J. C. (2006). *Angew. Chem. Int. Ed.* **45**, 7674–7715. 10.1002/anie.200602482 17115468

[bb8] Johnson, J. J. & Sames, D. (2000). *J. Am. Chem. Soc* **122**, 6321–6322.

[bb9] Nardelli, M. (1983). *Acta Cryst.* C**39**, 1141–1142.

[bb10] Sheldrick, G. M. (1996). *SADABS* University of Göttingen, Germany.

[bb11] Sheldrick, G. M. (2008). *Acta Cryst.* A**64**, 112–122. 10.1107/S0108767307043930 18156677

[bb12] Spek, A. L. (2009). *Acta Cryst.* D**65**, 148–155. 10.1107/S090744490804362X PMC2631630 19171970

[bb13] Staveren, D. R. V. & Metzler-Nolte, N. (2004). *Chem. Rev.* **104**, 5931–5986. 10.1021/cr0101510 15584693

